# The *Arabidopsis thaliana* Homeobox Gene *ATHB12* Is Involved in Symptom Development Caused by Geminivirus Infection

**DOI:** 10.1371/journal.pone.0020054

**Published:** 2011-05-18

**Authors:** Jungan Park, Hyun-Ju Lee, Choong-Ill Cheon, Sung-Han Kim, Yoon-Sun Hur, Chung-Kyun Auh, Kyung-Hwan Im, Dae-Jin Yun, Sukchan Lee, Keith R. Davis

**Affiliations:** 1 Department of Genetic Engineering, Sungkyunkwan University, Suwon, Korea; 2 Department of Biological Science, Sookmyung Women's University, Seoul, Korea; 3 Division of Life Sciences, Mokpo National University, Muan, Korea; 4 Department of Biology, University of Incheon, Incheon, Korea; 5 Division of Applied Life Science, Plant Molecular Biology and Biotechnology Research Center and Environmental Biotechnology National Core Research Center, Gyeongsang National University, Jinju, Korea; 6 Owensboro Cancer Research Program, Department of Pharmacology and Toxicology, School of Medicine, James Graham Brown Cancer Center, University of Louisville, Louisville, Kentucky, United States of America; Institute of Infectious Disease and Molecular Medicine, South Africa

## Abstract

**Background:**

Geminiviruses are single-stranded DNA viruses that infect a number of monocotyledonous and dicotyledonous plants. *Arabidopsis* is susceptible to infection with the Curtovirus, *Beet severe curly top virus* (BSCTV). Infection of *Arabidopsis* with BSCTV causes severe symptoms characterized by stunting, leaf curling, and the development of abnormal inflorescence and root structures. BSCTV-induced symptom development requires the virus-encoded C4 protein which is thought to interact with specific plant-host proteins and disrupt signaling pathways important for controlling cell division and development. Very little is known about the specific plant regulatory factors that participate in BSCTV-induced symptom development. This study was conducted to identify specific transcription factors that are induced by BSCTV infection.

**Methodology/Principal Findings:**

*Arabidopsis* plants were inoculated with BSCTV and the induction of specific transcription factors was monitored using quantitative real-time polymerase chain reaction assays. We found that the *ATHB12* and *ATHB7* genes, members of the homeodomain-leucine zipper family of transcription factors previously shown to be induced by abscisic acid and water stress, are induced in symptomatic tissues of *Arabidopsis* inoculated with BSCTV. *ATHB12* expression is correlated with an array of morphological abnormalities including leaf curling, stunting, and callus-like structures in infected *Arabidopsis*. Inoculation of plants with a BSCTV mutant with a defective *c4* gene failed to induce *ATHB12*. Transgenic plants expressing the BSCTV *C4* gene exhibited increased *ATHB12* expression whereas BSCTV-infected *ATHB12* knock-down plants developed milder symptoms and had lower *ATHB12* expression compared to the wild-type plants. Reporter gene studies demonstrated that the *ATHB12* promoter was responsive to BSCTV infection and the highest expression levels were observed in symptomatic tissues where cell cycle genes also were induced.

**Conclusions/Significance:**

These results suggest that *ATHB7* and *ATHB12* may play an important role in the activation of the abnormal cell division associated with symptom development during geminivirus infection.

## Introduction

Viral pathogens can induce dramatic morphological and developmental changes in plants [1], [2]. Geminiviruses are small, generally phloem-limited, ssDNA viruses with a genome size of 2.6–5.2 kb that infect a wide range of both monocotyledonous and dicotyledonous plants, including several economically important crop species [3]. *Beet curly top virus* (BCTV) and *Beet severe curly top virus* (BSCTV) are Curtoviruses, which are monopartite, leafhopper-transmitted viruses that infect only dicotyledonous plants. These two viruses are very similar in both genome structure and pathogenesis, but BSCTV causes more severe symptoms than BCTV [4], [5], [6]. The viral genome of BSCTV encodes seven open reading frames (ORFs). The protein products of these genes are involved in viral structure and insect vector transmission (capsid protein, V1), replication (C1), pathogenicity (C2) replication enhancement (C3), movement (V1, V3), ssDNA accumulation (V2), and symptom development (C4) [7], [8], [9], [10], [11].

In *Arabidopsis*, symptoms of BCTV and BSCTV infection can range from mild to severe and are dependent on the interactions between the virus strain and *Arabidopsis* ecotype that is infected [4], [5]. Symptoms in *Arabidopsis* include stunting of the plant, leaf curling, malformation of floral tissue, swelling of inflorescence bolts, anthocyanin production, and in some cases death of the plant [4], [5]. Because of its limited coding capacity, BSCTV must depend on the host cell to supply most of the proteins that are necessary for it to complete its life cycle, especially those proteins involved in the replication of viral DNA. BSCTV must therefore either infect cells already in S-phase or alter the state of the cells so that they are competent to synthesize DNA, a strategy employed by vertebrate RNA and DNA transforming viruses [12], [13], [14]. Several studies of the role of BCTV ORF C4 in symptom development and its potential interaction with host cell proteins have been completed [11], [15], [16], [17]. Mutations in BCTV ORF C4 have a pronounced effect on symptom development in several hosts, including *Nicotiana benthamiana* and *Beta vulgaris*. *N. benthamiana* plants infected with ORF *c4* mutant viruses showed greatly reduced or mild symptoms [17] whereas infected *B. vulgaris* plants were asymptomatic [11]. Expression of the BCTV and BSCTV C4 proteins in transgenic *Arabidopsis* results in phenotypes that mimic symptoms seen during viral infection [18], [19]. Taken together, these results demonstrate that C4 plays an important role in symptom development in plants and that the induction of aberrant cell division is a key component of BCTV- and BSCTV-induced developmental abnormalities.

It is currently not clear how geminivirus-encoded proteins interact with host components to induce the pathomorphogenic phenotypes associated with virus infection. Homeobox genes that are key regulators of plant development have been implicated to be involved in the process of pathomorphogenesis [20]. However, a direct correlation between pathomorphogenesis and homeobox genes has not yet been established. The *Arabidopsis* genome encodes a large number of homeobox genes that can be categorized into six major families [21]. A family unique to plants is the homeodomain-leucine zipper (HD-Zip) transcription factors that include both a homeodomain and a leucine zipper motif [22], [23], [24], [25], [26], [27], [28]. HD-Zip proteins are important regulators of plant development, including the integration of environmental stimuli with developmental pathways [21], [29].

*Arabidopsis* contains 47 HD-Zip genes that are grouped into four distinct subfamilies based upon gene structure and function. The expression of the *Arabidopsis* Class II *ATHB2* and *ATHB4* genes increases considerably after treatment with far-red-rich light [30]. The *ATHB7*(Class I) and *ATHB8* (Class III) genes are induced by exogenous plant hormones including abscisic acid (ABA) and auxin [31], [32], and *ATHB7* has been shown to be induced by drought [33], [34]. *ATHB12*, isolated from *A. thaliana* in 1998 [34] and characterized as a member of the Class I HD-Zip family [21], [33], is induced by ABA and water stress [33], [34], [35], [36], [37]. The *ATHB12* gene also is expressed in all organs of the plant and is similar to the paralogous gene, *ATHB7*. *ATHB12* and *ATHB7* share similarities in sequence, a common intron-exon organization, and similar specificities in DNA binding that distinguish the two paralogs from other Class I HD-Zip genes in *Arabidopsis* [34], [37] and appear to be key factors in the integration of development with abiotic stress.

In this study, we found that both *ATHB12* and *ATHB7* expression were induced by BSCTV infection of *Arabidopsis* and that BSCTV-induced symptom development was tightly correlated with the transcriptional activation of *ATHB12*. Infection of an *ATHB12* T-DNA insertion mutant demonstrated that *ATHB12* is necessary for complete symptom development. These results demonstrate that unique HD-Zip proteins are involved in the pathomorphogenesis caused by the biotic stress imposed on *Arabidopsis* during BSCTV infection and identifies a new class of host proteins that regulate host responses to virus infection.

## Results

### Expression of *ATHB12* in BSCTV-infected *Arabidopsis*

Since BCTV- and BSCTV-induced symptom development likely depends on changes in gene expression, we conducted a preliminary screen using DNA microarrays to compare the gene expression patterns in inflorescence tissues from mock-inoculated and BSCTV-infected plants. This preliminary screen suggested that *ATHB12* was strongly induced in symptomatic tissues of BSCTV-infected *Arabidopsis* plants and further studies focused on confirming this result.

**Figure 1:**
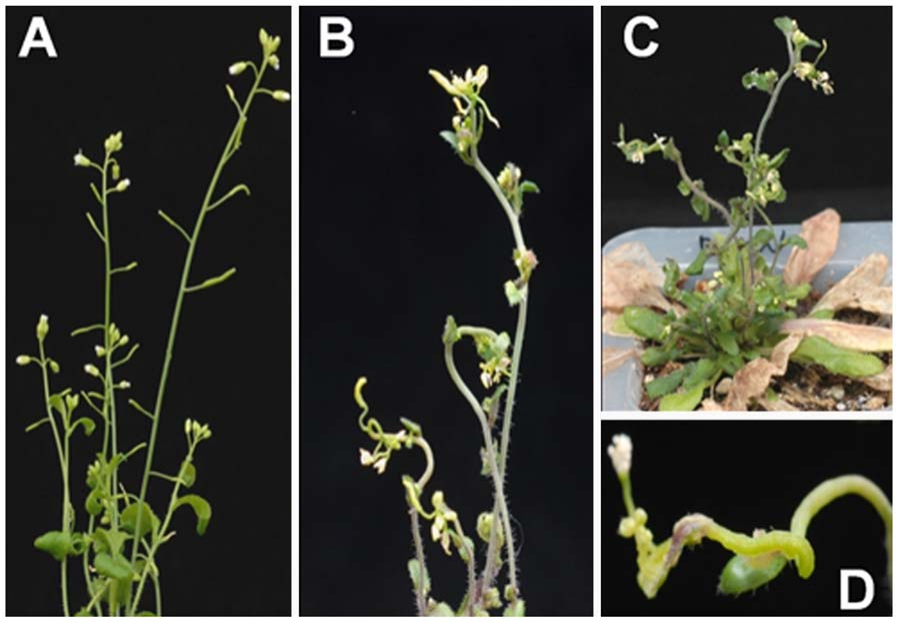
BSCTV-induced disease symptoms in *Arabidopsis*. BSCTV infection was accomplished by agroinoculation of the center of rosette leaves of 4-week old plants by pinpricking. (A) Mock-inoculated *Arabidopsis* developed normal flowers and siliques on the shoot tips. (B) Typical disease symptoms such as curling of shoot tips, siliques, and cauline leaves on BSCTV-infected inflorescences. Anthocyanin accumulated on stunted axillary buds or shoots with callus-like structures. (C) Stunted and curled inflorescence stems on a whole BSCTV-infected plant. (D) Magnified shoot tips with curved, swollen and callus-like structures.

**Figure 2:**
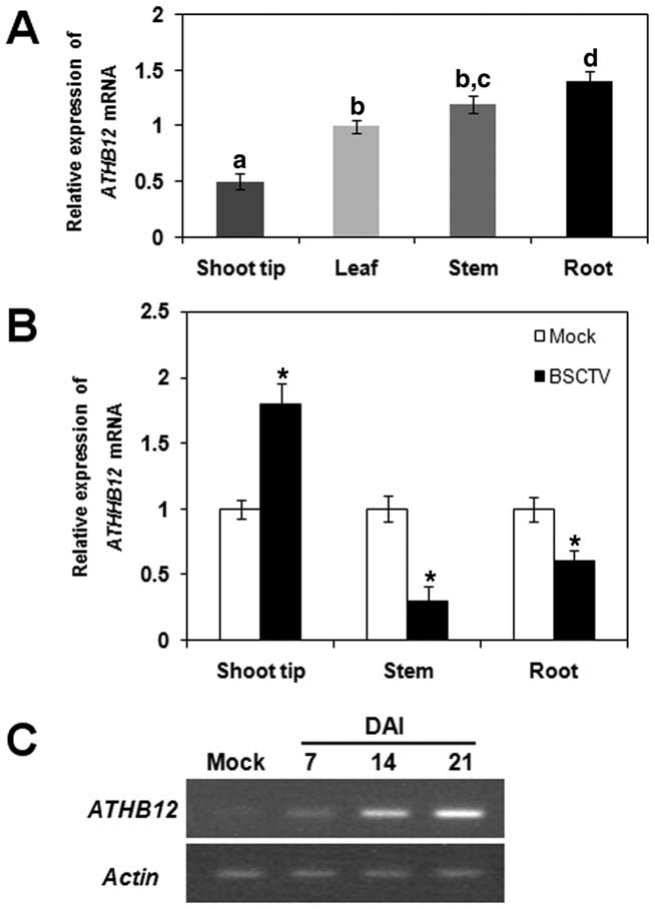
Comparison of *ATHB12* gene expression in uninfected and BSCTV-infected *Arabidopsis* plants. (A) *ATHB12* gene expression in inflorescence shoot tips, leaves, inflorescence stems, and roots of uninfected *Arabidopsis*. (B) *ATHB12* gene expression in BSCTV-infected plants showing significant induction in symptomatic inflorescence shoot tips. (C) Time course of *ATHB12* accumulation in response to BSCTV infection using semi-quantitative PCR. The data shown in (A) and (B) represent the mean ± S.D. of three or four experiments. Different letters (Panel A) or * (Panel B) refer to significant differences between mean values as determined using a Tukey's Multiple Comparison Test (P<0.05).

BSCTV-infected *Arabidopsis* showed the typical phenotypes of stunted inflorescence stems, leaf and stem curling, and malformation of inflorescence shoot tip and axillary bud structures caused by abnormal cell division (Figure 1). Quantitative real-time polymerase chain reaction (RT-PCR) assays revealed that *ATHB12* transcript levels were lowest in the inflorescence shoot tip and highest in the root in uninfected plants (Figure 2A). BSCTV infection significantly modulated the transcript levels of *ATHB12* in different organs within three weeks post-inoculation (Figure 2B). *ATHB12* transcript levels in the inflorescence shoot tip were approximately 2-fold higher in BSCTV-infected plants compared to mock-inoculated plants. At the same time, *ATHB12* expression in the inflorescence stems or roots of BSCTV-inoculated plants was reduced 2–4 fold. Analysis of *ATHB12* transcript levels using semi-quantitative PCR detected gene expression 7 days after BSCTV infection in the shoot tips of *Arabidopsis*, and expression continued to rise for up to 21 days in parallel with increasing symptom development (Figure 2C).

### Activation of *ATHB12* promoter activity by BSCTV infection

**Figure 3:**
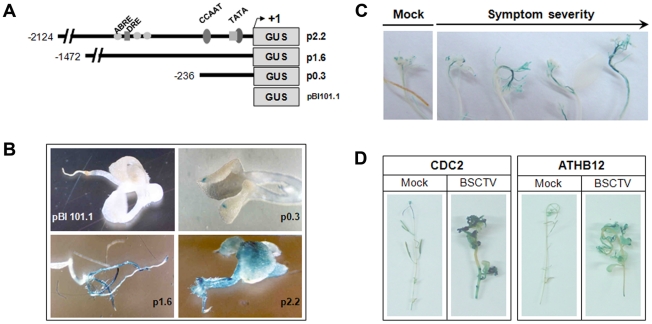
Localization of GUS activity in *Arabidopsis* plants expressing *ATHB12* promoter-*gusA* fusion or *CDC2* promoter-*gusA* fusion constructs. (A) Diagram depicting the three *ATHB12* promoter fusions used for these studies. The positions of several specific known transcription binding sites are shown. (B) GUS staining of representative plants expressing the three *ATHB12* promoter-*gusA* fusions. Note that the p2.2 line showed systemic GUS staining throughout the seedlings, whereas the p1.6 and p0.3 lines only stained positive in the roots and cotyledons, respectively. Based on this result we utilized the p2.2 line for BSCTV infection studies. (C) GUS staining of BSCTV-infected plants. All infected plants had a high intensity of GUS staining in the symptomatic tissues in the inflorescence shoot tip where aberrant cell division was evident in response to BSCTV infection. (D) Comparison of *ATHB12* and *CDC2* promoter activity in BSCTV-infected plants. All infected plants showed significant GUS staining in the symptomatic inflorescence shoot tips, demonstrating a correlation of *ATHB12* induction with the activation of *CDC2*. The pBI101.1 line was used for a negative control in all studies and was always negative for GUS activity.

To determine if the increased *ATHB12* transcript levels observed after BSCTV infection were due to transcriptional activation, we utilized a reporter gene approach based on histochemical staining of transgenic plants expressing an *ATHB12* promoter-*gusA* fusion. Figure 3A depicts the constructs used in these experiments. Three different putative promoter regions comprised of 236, 1,472, and 2,124 bp of the 5′ upstream region of the *ATHB12* gene transcriptionally fused to the *E. coli gusA* gene encoding β-glucuronidase (GUS) [38] were introduced into *Arabidopsis*. Evaluation of GUS activity in uninoculated seedlings demonstrated that GUS activity increased depending on the size of the *ATHB12* promoter (Figure 3B). The shortest promoter (p0.3) exhibited very little expression which was localized to discreet regions at the tips of the cotyledons. The 1,472 bp (p1.6) promoter conferred significant expression in the roots, but not in the shoot. The 2,124 bp promoter (p2.2) conferred the highest level of GUS activity in seedlings, with significant expression in both the root and the shoot. To test the effects of BSCTV infection on *ATHB12* promoter activity, transgenic plants containing the p2.2 promoter were inoculated with BSCTV. Histochemical localization of GUS activity was examined in inflorescence stems from plants showing varying levels of symptom develop ranging from moderate to severe. The intensity of GUS activity correlated with the severity of symptoms in the shoot tips (Figure 3C). To determine if *ATHB12* promoter activity was associated with induction of cell cycle genes, similar studies were conducted with transgenic *Arabidopsis* plants that expressed a reporter gene composed of the *CDC2a* promoter fused to the *gusA* gene [39]. Histochemical staining for GUS activity showed that cells in the symptomatic tissues at the shoot tip of the transgenic plants expressed high GUS activity and the overall pattern of expression was similar to that observed in the *ATHB12-gusA* plants (Figure 3C). In mock-inoculated control plants, *CDC2a* and *ATHB12* promoter activity was limited to the elongation region of the inflorescence stem and in developing flower buds at three weeks after inoculation (Figure 3C and 3D).

### BSCTV-induced expression of *ATHB12* requires C4

**Figure 4:**
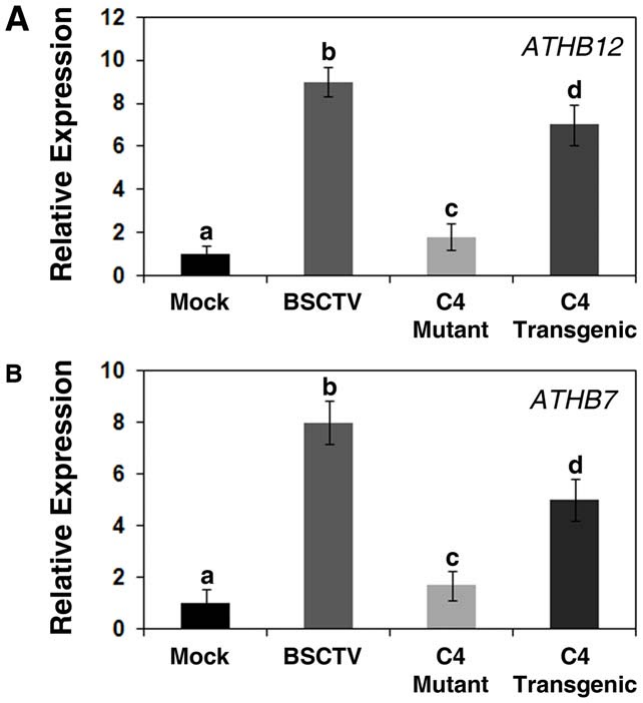
Expression of *ATHB12* and *ATHB7* in plants infected with wild-type BSCTV or a BSCTV *c4* mutant. Quantitative RT-PCR was used to determine the relative expression levels of *ATHB12* in the inflorescence shoot tips of infected plants and mock-inoculated Col-O compared to the transcript levels observed in a transgenic line expressing BSCTV C4. Note that both *ATHB12* and *ATHB7* transcript levels are significantly induced by wild-type BSCTV whereas no significant induction was seen in plants inoculated with the *c4* mutant virus. Transgenic *Arabidopsis* that constitutively expressed BSCTV C4 exhibited significantly higher expression of *ATHB12* and *ATHB7* than wild-type mock inoculated plants. The data shown represent the mean ± S.D. of three or four experiments; different letters above the bars refer to significant differences between mean values as determined using a Tukey's Multiple Comparison Test (P<0.05).

Previous studies in *N. benthamiana* and *B. vulgaris* suggest that the C4 gene is required for symptom development in BCTV-infected plants. To determine if the BSCTV C4 gene was required for the induction of *ATHB12*, *ATHB12* accumulation was compared in BSCTV-infected plants, BSCTV *c4* mutant-infected plants, and transgenic plants expressing the BSCTV C4 gene under the control of the *Cauliflower mosaic virus* (CaMV) 35S promoter (Figure 4A). The expression of *ATHB12* increased significantly in BSCTV-infected plants and transgenic *Arabidopsis* constitutively expressing the BSCTV C4 gene. In contrast, the level of *ATHB12* increased only slightly in BSCTV *c4*-mutant infected *Arabidopsis*. Since previous studies have shown that *ATHB7* is co-regulated with *ATHB12* in response to ABA and water stress, we also examined the expression of *ATHB7* in this experiment (Figure 4B). The expression pattern of *ATHB7* was very similar to that observed for *ATHB12*; *ATHB7* transcripts were higher in BSCTV infected plants and transgenic lines expressing C4 whereas plants inoculated with the BSCTV *c4* mutant showed little induction.

To determine if *ATHB12* expression is required for symptom development in BSCTV-infected plants, an *ATHB12* knock-down (KD) mutant [40] and the wild-type background ecotype Ws-O were inoculated with BSCTV. Inoculated plants were then assessed for symptom severity. *ATHB12* KD plants developed fewer symptoms in response to BSCTV infection (Table 1). Of the inoculated *ATHB12* KD plants, 14% were asymptomatic, 44% had mild symptoms and 42% developed severe symptoms. In contrast, a larger number of inoculated wild-type ecotype Ws-O plants displayed severe (68%) and mild symptoms (31%) and only 1% of Ws-O plants were asymptomatic after BSCTV infection.

**Table 1:** Symptom severity and infectivity of BSCTV in the *ATHB12* KD and Ws-O.

	Symptom Severity		
	aAsymptomatic	bMild Symptoms	cSevere Symptoms
**Plant Line**	α/N (%)	α/N (%)	α/N (%)
Ws-O	1/74 (1)	23/74 (31)	50/74 (68)
*ATHB12* KD	10/71 (14)	31/71 (44)	30/71 (42)

a Asymptomatic: Inoculated plants were indistinguishable from mock-inoculated plants and inflorescence bolts did not exhibit any abnormal structures.

b Mild symptoms: Mild curling of inflorescence shoot tips and inflorescence bolt height was comparable to control plants.

c Severe symptoms: Severe curling of bolts, malformed inflorescence structures, and severe stunting of plants.

## Discussion

Since geminiviruses have a very compact genome that does not encode essential replication proteins, they are highly dependent on the recruitment of plant-host factors to replicate and complete their life cycle [41]. Earlier studies have shown that *Tomato golden mosaic virus* (TGMV) induces the transcription and accumulation of proliferating cell nuclear antigen (PCNA) in cells that normally do not express PCNA [42], [43]. These studies provided one of the first indications that geminiviruses have the ability to reprogram plant cells to support viral replication. A more recent study demonstrates that geminivirus expression can affect the expression of over 5,000 genes [44]. The BCTV/BSCTV interaction with *Arabidopsis* provides an excellent system to study this reprogramming since in this case, viral infection leads to the induction of the cell cycle and aberrant cell divisions [4], [5], [18], [19], [45]. How BCTV/BSCTV infection results in aberrant cell division is not clear, however it is likely that it requires the activation of specific genes in infected cells. RKP, a putative RING finger E3 ligase protein with homology to the human cell cycle regulator KPC1 has been shown to be induced by the BSCTV symptom determinant C4 [15]. The BCTV and BSCTV C4 proteins have been shown to interact with *Arabidopsis* shaggy-related protein kinases [16], [46] and the AL1 protein interacts with the kinases GRIK1 and GRK2 [47], suggesting that effects on key signal transduction pathways may be important in symptom development. Similarly, the BCTV and *Spinach curly top virus* (SCTV) C2 proteins and the TGMV AL2 protein interact with Arabidopsis AKIN11, a SNF1-related kinase, and adenosine kinase 2 (ADK2) [48], [49], [50]. Interactions have also been identified that appear to affect hormonal regulation of plant cell division and development. The *Tomato yellow leaf curl China virus* (TYLCCNV) βC1 protein has been shown to interact with ASYMMETRIC LEAVES 1 (AS1) and to partially suppress JA-controlled responses [51]. The TGMV AL2 and SCTV C2 proteins have recently been shown to induce cytokinin-responsive genes [52]; this may be a result of the previously described interaction with ADK2 [48]. This is consistent with other studies showing that geminivirus-induced symptoms may involve changes in hormone signaling [1], [5], [45].

Our current study demonstrates that the *ATHB7* and *ATHB12* homeobox genes that are known to be regulated by ABA and water stress [33], [34], [35] are induced in *Arabidopsis* by BSCTV infection. The induction of these homeobox genes was correlated with the induction of aberrant cell division and the development of severe symptoms such as leaf curling, stunting, and formation of callus-like structures. Many plant homeobox genes are related to hormonal signaling [31], [33], [34], [53], [54] and alteration of hormonal balance is a well known feature of a number of plant-pathogen interactions [55]. BCTV and BSCTV induces typical symptoms similar to those caused by an imbalance of auxin concentration in symptomatic tomato and *Arabidopsis* tissues and is correlated with increased promoter activity of auxin-induced genes [1], [5], [45]. BSCTV induction of *ATHB7* and *ATHB12* is not likely due to effects on auxin. Previous studies have shown that hormonal control of these genes is regulated primarily by ABA in *Arabidopsis* [33], [34], [35], which is consistent with our studies of p2.2-*gusA* transgenic plants treated with auxin that showed auxin does not induce *ATHB12* promoter activity (data not shown). The induction of *ATHB12* and *ATHB7* suggests that BSCTV infection also may alter ABA levels or that there is an ABA-independent mechanism for activating *ATHB7* and *ATHB12* expression. Other studies [16] have shown that the BCTV C4 protein binds to *Arabidopsis* shaggy-related kinase AtSKη, a negative regulator of the brassinosteroid signaling pathway, although there is no evidence that brassinosteroid levels are affected by BSCTV infection. We found that brassinosteroid treatment of wild-type seedlings or *ATHB12*-promoter-*gusA* transgenic plants did not induce *ATHB12* transcripts or GUS expression, respectively (data not shown). Thus, the induction of *ATHB12* expression probably does not directly involve brassinosteroids.

A role for *ATHB12* induction in symptom development is supported by our studies of transgenic *Arabidopsis* expressing the BSCTV C4 gene and the *ATHB12* KD line. The BSCTV ORF C4 gene is an important factor in symptom development [11], [17] and C4-expressing transgenic plants show systemically abnormal development that is similar to BSCTV-induced symptoms in inflorescence shoot tips. We found that the expression level of *ATHB12* was correlated with the severity of abnormal development in C4 transgenic plants and that infection of *Arabidopsis* with a BCTV *c4* mutant did not induce significant symptom development or *ATHB12* gene expression, implying a relationship between the functions of the BSCTV C4 protein and the effects of *ATHB12* induction on symptom development. This is consistent with the observation that BSCTV-infected *ATHB12* KD plants with reduced *ATHB12* transcript levels exhibited significantly milder symptoms than wild-type plants. The fact that the *ATHB12* KD plants exhibited some symptom development is likely due to the fact the KD plants express approximately 20–30% of normal levels of *ATHB12* transcripts and that *ATHB7* expression is not affected in the KD plants. Taken together, our data suggest that the *ATHB7* and *ATHB12* genes may be important factors in BSCTV-induced symptom development. This is particularly interesting since these two homeobox genes have previously been found to be important in coordinating abiotic stress signals with growth and development [21], [29]. The fact that *ATHB7* and *ATHB12* are induced by BSCTV infection suggest that alterations in ABA biosynthesis or localization may be involved in symptom development. Further genetic and biochemical studies using mutant *Arabidopsis ATHB7* and *ATHB12* plants, hormone metabolism mutants, and BSCTV mutants are needed to determine the precise connection between BSCTV C4 and *ATHB7*/*ATHB12* induction with the activation of abnormal cell division that is associated with BSCTV-induced symptom development.

## Materials and Methods

### Plant growth and virus inoculation

*Arabidopsis* ecotype Col-0 was obtained from the *Arabidopsis* Biological Resource Center at the Ohio State University. The pCDC2 transgenic line, which was made by fusion of the *CDC2* promoter with the *gusA* gene, was provided by Dr. Von Montague [39] and the transgenic Col-0 constitutively expressing BSCTV ORF C4 was previously described [19]. The *ATHB12* KD mutant [40] was kindly provided by Dr. Cheon, Sookmyung Women's University, Korea. Seeds were planted in flats containing artificial soil, covered with plastic domes, and then placed and maintained in growth chambers at 18–22°C and 50–80% relative humidity, with a day length of 10 or 12 hrs from fluorescent bulbs at an intensity of 100–200 uE·m^−2^·s^−1^ [4]. Plastic domes were removed 10–14 days after sowing when seedlings were well established. Four- to five-week-old plants were inoculated with infectious viral DNA by agroinoculation of wounds produced in the crown of the rosette by needle puncture, as previously described [4], [56]. Control mock-inoculated plants were agroinoculated with *Agrobacterium tumefaciens* GV3101 haboring an empty pMon521 vector.

### BSCTV strain

The isolation and characterization of full-length infectious clones of BSCTV has been previously described [6]. The BSCTV clone was provided by Dr. Drake Stenger (Agricultural Research Service, United States Department of Agriculture, Parlier, California, USA) and the BSCTV leftward ORF C4 mutant clone was provided by Dr. Kenneth Buckley (Ohio State University, Columbus, Ohio, USA).

### Construction of *ATHB12* promoter-*gusA* transgenic *Arabidopsis lines*

A putative full-length promoter that is responsive to ABA [34], [35] was generated by constructing a transcriptional fusion of the 2.1 kb-5′ upstream of *ATHB12* to the β-glucuronidase reporter gene (*gusA*) in vector pBI121 [57]. To define the possible location of the *cis*-acting region responsible for BSCTV infection, smaller segments of this 2.1 kb region were generated by PCR and fused with GUS. All vectors were confirmed by DNA sequencing and transferred to *A. tumefaciens* strain GV3101. The resulting *A. tumefaciens* strains were used to transform *Arabidopsis* plants as described [19]. The following primer sequences were used: p2.2, forward: 5′-GAA TCT CTG AGT TGC TGA TAT TGG C-3′ and reverse: 5′-GGC TTT CTT A AT GGT GCC AA A TGG C-3′; p1.6, forward: 5′-GAA TCT CTG AGT TGC TGA TAT TGG C-3′ and reverse: 5′-TTG ACT AAT AAA AGT ATC GCA AGG C-3′; p0.3, forward: 5′-GAA TCT CTG AGT TGC TGA TAT TGG C-3′ and reverse: 5′-ATG GCT TAA GGA TAA AGT GAC GTT G-3′; and p0.2, forward: 5′-GAA TCT CTG AGT TGC TGA TAT TGG C-3′ and reverse: 5′-CTA CTT GTC TGA AAT GTC TAC AGA T-3′.

### Histochemical staining for GUS activity

Tissues were stained for GUS activity in solutions containing 100 mM sodium phosphate, pH 7.0, 10 mM EDTA, 0.1% Triton X-100, 1 mg/mL 5-bromo-4-chloro-3-inolyl-β-D-GlcUA, cyclohexylammonium salt (X-Gluc), 100 µg/mL chloramphenicol, 2 mM potassium ferricyanide, and 2 mM potassium ferrocyanide, as previously described [58]. In brief, tissues were harvested directly into a volume of staining solution sufficient to cover the tissue and placed under a house vacuum for 10 min. Staining was carried out at 37°C in the dark for 48 hr. After staining, the tissue was incubated in 70% ethanol to remove the chlorophyll. The ethanol was changed several times until the tissue was clear.

### Total RNA isolation and quantitative RT-PCR

RNA isolation and analysis was performed as described by [59]. To analyze the quantitative expression pattern of each gene by RT-PCR, the cDNA strand was synthesized from 2 µg of total RNA using oligo (dT) primers and Moloney murine leukemia virus (MMLV) reverse transcriptase (SuperBio Co., Korea). Primers used for the quantitative RT-PCR were designed by Primer3 (http://www-genome.wi.mit.edu/cgi bin/primer/ primer3.cgi/ primer3_www.cgi) and synthesized by Bioneer (Bioneer Co., Korea). The primers used were *ATHB12*, forward: 5′-GGTTAGACCAAGGGAGTGTTCTATGT-3′ and reverse: 5′-CAATTCTCAGAAGATGTCAAGCAACT-3′; *ATHB7*, forward: AGATGAAAGATGATAGGGGTCATCA and reverse: ACAACTATCAGCTGGTTCAACAATG. Quantitative RT-PCR amplification was done using the modification of Schefe et al. [60]. PCR conditions using a Rotor-Gene 3000 (Corbett Research Co., Australia) included 30 cycles of 10 sec denaturation at 94°C, 15 sec annealing at 60°C, and 20 sec polymerization at 72°C. The products of quantitative RT-PCR assays were analyzed by 1% agarose gel electrophoresis and showed a single band with the size predicted in the template sequence. Two negative controls, one each without the total RNA template or reverse transcriptase, were included in each experiment. The housekeeping *Arabidopsis actin2* gene (U41998) was used as an internal normalization standard to quantify the target mRNAs (forward: 5′GAAAAGATCTGGCATCACACTTTCTA3′ and reverse: 5′ACATACATAGCGGGAGCGTTAAAGG3′). Each data point represents the average of three or four experiments and the error bars represent the standard deviation. Statistical analyses were done using GraphPad Prism (GraphPad Software, USA) and included a one-way ANOVA with a Tukey's Multiple Comparison Test for significant differences.

For semi-quantitative PCR, cDNA synthesis was done as described for RT-PCR and the subsequent PCR reactions with a gene-specific primer set were carried out with the following cycling conditions: 94°C for 5 min, 25 cycles of 94°C for 1 min, annealing temperature as specified for 1 min, and 72°C for 1–2 min, with a single final extension at 72°C for 5 min. The *actin2* gene was used as an internal control. Gene expression patterns between mock and infected groups were compared following electrophoresis on 1.5% agarose gels.
